# A Case of Mucoepidermoid Carcinoma of the Larynx

**DOI:** 10.7759/cureus.34455

**Published:** 2023-01-31

**Authors:** James Philip Skliris, Katerina Marini, Alexandros Poutoglidis, Ioannis Matzarakis, Paraskevi Karamitsou

**Affiliations:** 1 Department of Pathology, George Papanikolaou General Hospital, Thessaloniki, GRC; 2 Department of Otorhinolaryngology-Head and Neck Surgery, G. Gennimatas General Hospital, Thessaloniki, GRC; 3 Department of Otorhinolaryngology-Head and Neck Surgery, George Papanikolaou General Hospital, Thessaloniki, GRC

**Keywords:** tumor, larynx, mucoepidermoid carcinoma, otolaryngology, oncology, cancer

## Abstract

Mucoepidermoid carcinoma is a common malignant neoplasm of the salivary glands. While quite common in the oral cavity, it is rare in the larynx. A middle-aged male patient presented to the otolaryngology clinic of our institution with the chief complaint of hoarseness. A supraglottic subepithelial mass was detected on the left laryngeal ventricle after a comprehensive clinical examination. Eventually, the diagnosis was established with a biopsy after a direct laryngoscopy. The multidisciplinary team of our institution suggested total laryngectomy without adjuvant modalities. An uneventful procedure followed and the patient remains free of disease and up to date. Mucoepidermoid tumors of the larynx are rare and surgical treatment is strongly indicated as the treatment of choice.

## Introduction

Squamous cell carcinoma (SCC) is the most dominant head and neck cancer and is responsible for more than 90% of laryngeal cancers [1]. Locally advanced SCC of the larynx can be treated either with radical surgery or with combined radiotherapy and chemotherapy for organ preservation purposes [2]. On the other hand, mucoepidermoid carcinoma (MEC) is a common salivary gland tumor. Despite being quite common in the parotid gland and oral cavity, it is quite rare in the larynx and accounts for less than 0.5% of all malignant laryngeal tumors [3]. Adenoid cystic cancer of the larynx is the most common neoplasm of minor salivary gland origin [4]. Non-SCC of the larynx is a rare and challenging entity with no global consensus for its management. Our study aimed to raise awareness for diagnosing and treating laryngeal MEC.

## Case presentation

**Figure 1:**
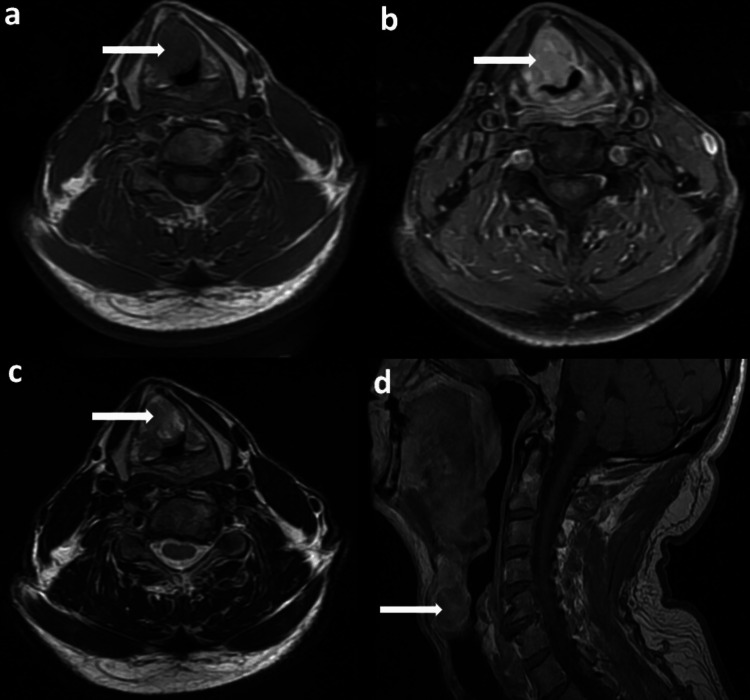
Magnetic resonance imaging of the neck after paramagnetic contrast enhancement. A laryngeal tumor (arrow) (30x20x22 mm) with no invasion of the surrounding structures. MRI (a) axial (T1W1 sequence), (b) axial (T1W1 sequence with fat-sat), (c) axial (T2W1 sequence), and (d) sagittal (T1W1 sequence).

A 62-year-old male presented to our ear, nose, and throat (ENT) outpatient clinic with the chief complaint of hoarseness for two months. His medical history was unremarkable for other conditions. He was a smoker (1 pack/year) for 35 years and social drinker. Flexible endoscopy revealed a solid, submucosal supraglottic mass. The mass was not friable and was located on the left side of the laryngeal ventricle. Magnetic resonance imaging (MRI) demonstrated a relatively circumscribed laryngeal tumor, measuring 30x20x22 mm, with disparate T2-weighted and medium to high T1-weighted signals after intravenous paramagnetic contrast agent enhancement. No invasion or infiltration of surrounding structures was recorded (Figures 1a-1d).

**Figure 2:**
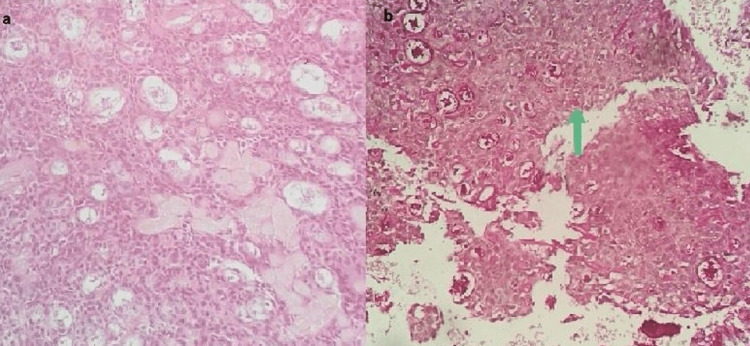
Histopathological examination of the patient. (a) Glandular formations are visible (H&E stain, 200x). In MEC, three following neoplastic cell types are characteristic: epidermoid, mucous, and intermediate, with the latter prevailing in high-grade cases. (b) Mucous cells are highlighted (arrow; PAS histochemical stain, 200x). PAS: periodic acid-Schiff; MEC: mucoepidermoid carcinoma

**Figure 3:**
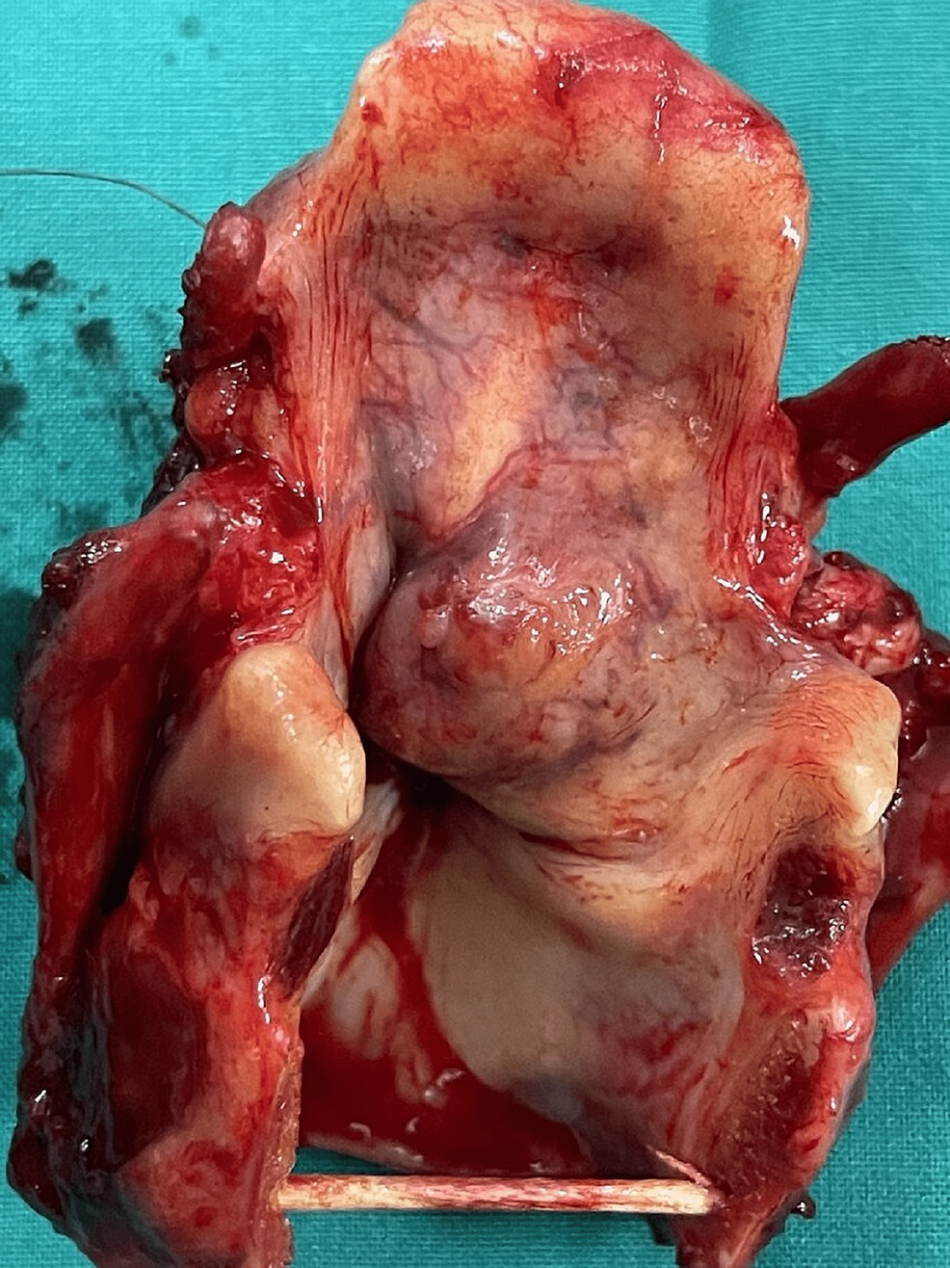
Surgical specimen with a large submucosal mass in the left laryngeal ventricle.

The patient underwent a panendoscopy under general anesthesia, and biopsies indicated an intermediate-grade mucoepidermoid carcinoma of the left laryngeal ventricle (Figures 2a, 2b). Further imaging with neck and thorax computed tomography (CT) was negative for a second primary tumor or secondary metastatic disease. Therefore, he was staged as clinical cT2N0M0. The multidisciplinary team of our institution recommended a total laryngectomy due to the location and the histopathologic characteristics of the tumor (Figure 3). The patient underwent an uneventful total laryngectomy and was discharged on the 11th postoperative day. The tumor was excised in negative margins and no adjuvant treatments were recommended. The final pathological staging was also pT2N0M0. The closed monthly follow-up is clear and up to date with no recurrence.

## Discussion

Minor salivary gland carcinomas are usually found in the oral cavity and especially in the hard palate [3]. MEC of the major salivary glands was first described in 1924. However, the first case of laryngeal MEC was recorded 39 years later in 1963. Although MEC etiology remains unknown, exposure to asbestos, lead, alcohol, viral infections, ionizing rays, and genetic factors are considered predisposing factors [5]. MECs share a definite gender preference with a man-to-woman ratio measured at 9:1 and possibly arise from the excretory ducts of seromucous glands. The mean age of diagnosis is 55 years and only a few cases of children or adolescents have been reported [4-8]. Laryngeal MECs affect the supraglottis (60%), followed by the glottis (30%) and the subglottis (10%) [3,7]. Because they tend to be developed submucosally, symptoms are usually mild, rendering early diagnosis difficult. In later stages, the manifestations are strongly related to the affected area. Supraglottic MECs generally cause dysphagia or even secondary otalgia, while glottic MECs appear with hoarseness or even stridor [3,7,9].

Due to their submucosal nature laryngoscopy often fails to identify early lesions [5]. Imaging is valuable in determining the stage of disease and planning the appropriate treatment modality. It is important to highlight that 50% of cases are presented with positive cervical lymph nodes or visceral metastases, especially in the lungs. Positron emission tomography (PET) is considered an excellent tool to detect distant metastasis. However, it should be interpreted with caution due to a high proportion of false-positive cases [10].

Definite diagnosis is based on histopathology and three main cellular subtypes have been described as follows: (a) epidermoid, (b) mucous, and (c) intermediate. Different proportions of cystic versus solid elements, as well as mitosis, necrosis, anaplasia, and perineural invasion, define the tumor grade as low, medium, and high, with the latter being more aggressive with greater metastatic potential. Differential diagnosis includes other types of salivary gland carcinomas like adenoid cystic carcinoma and SCC [7]. Poor prognostic factors include advanced stage and grade, positive margins of resection, cervical lymph node metastasis, pain, advanced age, and the prevalence of epidermoid cells [7,11,12].

As for treatment, total laryngectomy is the gold standard modality, with partial laryngectomy being also an option for low-grade, localized cases accompanied by neck dissection in all high-grade tumors or patients with apparent neck involvement [3]. Local recurrence is not rare, reaching around 50% in medium and high-grade tumors [3]. The role of adjuvant radiotherapy is yet unclear, although it is agreed to be implemented in advanced-stage or high-grade cases, particularly with perineural invasion. Other possible indications include positive surgical margins, medium grade, and nodal disease [7]. Five-year survival is dependent on histologic grade with 0-45% in high grade, 60-90% in medium grade, and 90-100% in low grade [4].

## Conclusions

This case highlights that clinical awareness for laryngeal MEC should be prompted. In the case of a submucosal laryngeal mass in a patient without the predisposing factors of SCC, head and neck cancer should be suspected for a neoplasm of salivary gland origin. The role of radiotherapy and chemotherapy (if any) is limited and should be considered only as adjuvant treatment. Surgical treatment is the gold standard treatment.

## References

[1] Tsetsos N, Poutoglidis A, Vlachtsis K, Stavrakas M, Nikolaou A, Fyrmpas G (2021). Twenty-year experience with salvage total laryngectomy: lessons learned. J Laryngol Otol.

[2] Obid R, Redlich M, Tomeh C (2019). The treatment of laryngeal cancer. Oral Maxillofac Surg Clin North Am.

[3] Nielsen TK, Bjørndal K, Krogdahl A, Primdahl H, Kristensen CA, Andersen E, Godballe C (2012). Salivary gland carcinomas of the larynx: a national study in Denmark. Auris Nasus Larynx.

[4] Vardaxi C, Skalias A, Karamitsou P, Forozidou E, Poutoglidis A (2022). Four years of disease-free survival after conservative treatment of subglottic adenoid cystic carcinoma. Cureus.

[5] Alimoglu Y, Mamanov M, Kaytaz A (2011). High-grade mucoepidermoid carcinoma of the larynx. J Craniofac Surg.

[6] Mokhtari S, Mokhtari S (2012). Clinical features and differential diagnoses in laryngeal mucoepidermoid carcinoma. Clin Med Insights Pathol.

[7] López F, Williams MD, Skálová A (2017). How phenotype guides management of the most common malignant salivary neoplasms of the larynx?. Adv Ther.

[8] Tanaka H, Kohno A, Kawabata K, Sato Y (2010). Mucoepidermoid carcinoma of an adolescent epiglottis. Jpn J Radiol.

[9] Forozidou E, Tsetsos N, Karamitsou P (2022). A rare cause of secondary otalgia. Ear Nose Throat J.

[10] Tsetsos N, Poutoglidis A, Arsos G, Tsentemeidou A, Kilmpasanis A, Katsampoukas D, Fyrmpas G (2022). (18)F-FDG-PET/CT interpretation pitfalls in patients with head and neck cancer. Am J Otolaryngol.

[11] Richards HW, Bertelsen C, Hamilton B, Sauer D, Schindler J (2022). Mucoepidermoid carcinomas of the larynx. Ann Otol Rhinol Laryngol.

[12] Mimica X, Yuan A, Hay A (2021). Mucoepidermoid carcinoma: evaluating the prognostic impact of primary tumor site. Oral Oncol.

